# Analysis of the status of perceived professional benefits among nursing students and their relationship with teaching behaviors among nursing teachers

**DOI:** 10.3389/fmed.2026.1736455

**Published:** 2026-04-30

**Authors:** Peilan Xu, Xiumin Zhang, Nan Wang, Caihong Wang

**Affiliations:** 1 Xinjiang Second Medical College, Karamay, Xinjiang, China; 2 Department of Nursing, People’s Hospital of Xinjiang Uygur Autonomous Region, Urumqi, Xinjiang, China; 3 Center of Respiratory and Critical Care Medicine, People’s Hospital of Xinjiang Uygur Autonomous Region, Urumqi, Xinjiang, China

**Keywords:** clinical clerkship, cross-sectional studies, internship, nursing, professional role

## Abstract

**Background:**

Clinical internship is a critical stage for shaping nursing students’ professional attitudes and identity. Perceived professional benefit during internship is associated with career satisfaction and retention; however, its relationship with clinical instructors’ teaching behaviors remains insufficiently described.

**Methods:**

A cross-sectional survey was conducted among undergraduate nursing interns in two tertiary hospitals in China. The Nurses’ Perceived Professional Benefit Questionnaire and the Nursing Clinical Teacher Effectiveness Inventory (NCTEI) were administered. A total of 387 valid questionnaires were analyzed. Descriptive analyses, Pearson/Spearman correlations, and multiple linear regression were performed.

**Results:**

Nursing students reported moderate-to-high perceived professional benefit, with the highest scores in self-growth and team belongingness. All NCTEI dimensions were positively associated with perceived professional benefit (*p* < 0.001), with stronger associations observed for instructors’ personal traits and interpersonal relationships. In the regression model, perceived professional benefit was lower among only-child students (β = −2.522, *p* = 0.002) and higher among students who voluntarily selected nursing as a major (β = 3.374, *p* < 0.001) and those reporting higher ratings of teachers’ personal traits (β = 4.782, *p* < 0.001).

**Conclusion:**

In this cross-sectional sample, clinical teaching behaviors—particularly instructors’ personal traits—were associated with nursing interns’ perceived professional benefit. These findings support strengthening clinical teacher development (especially interpersonal/mentoring competencies) and enhancing career guidance to foster positive professional experiences during internship.

## Introduction

Nursing students constitute the primary reserve force of the future nursing workforce (1). The clinical internship period is a pivotal stage during which students, under the supervision of clinical nursing instructors, begin to assume nursing responsibilities and apply theoretical knowledge to real-world practice (2, 3). During this stage, students’ perceived professional benefit—their perceived positive gains from the nursing profession in terms of growth, value, and belonging—may shape professional attitudes and willingness to remain in nursing (4).

Clinical instructors play a central role in the clinical learning environment (5). Their teaching behaviors can influence how students adapt to the workplace, experience support, and develop confidence and professional belonging. From a humanistic education perspective, learner-centered teaching that respects students’ needs, encourages autonomy, and provides supportive interpersonal interactions may facilitate positive professional experiences and strengthen students’ recognition of nursing’s value (6). In this framework, dimensions such as instructors’ interpersonal relationships, professionalism, and personal traits can be viewed as key mechanisms through which the clinical learning environment affects students’ perceptions of professional benefit (7).

Although prior studies have examined nursing students’ professional attitudes and related constructs (e.g., professional identity), evidence remains limited regarding how specific clinical teaching behaviors are associated with perceived professional benefit during internship, and few studies have addressed this relationship in China (8, 9). This is an important gap because the clinical internship in China often occurs in high-intensity tertiary hospital settings (10, 11), where workload pressures and hierarchical clinical training structures may shape students’ learning experiences and professional perceptions (12, 13).

Therefore, this study aimed to describe the level of perceived professional benefit among undergraduate nursing interns in China and examine the associations between perceived professional benefit and clinical instructors’ teaching behaviors. The findings may inform targeted improvements in clinical teaching and mentoring practices and support strategies to foster positive professional experiences during internship.

## Materials and methods

### Study design

This study used a cross-sectional questionnaire survey design. Data were collected from undergraduate nursing interns during their clinical internship in two comprehensive tertiary (Grade A) teaching hospitals in Xinjiang, China. The study aimed to describe the level of perceived professional benefit among nursing interns and examine its associations with clinical instructors’ teaching behaviors. The reporting of this observational study was informed by the STROBE principles for cross-sectional studies.

### Participants

This study targeted undergraduate nursing students undergoing clinical internship in China. Eligible participants were full-time undergraduate nursing students admitted through the national unified college entrance examination system and entering clinical internship for the first time. Students from adult education programs, open education, top-up programs (e.g., RN-to-BSN), or those with prior clinical work experience were excluded. Participants were recruited using a convenience sampling method from two comprehensive tertiary (Grade A) teaching hospitals in Xinjiang, China (Urumqi and Karamay), both of which have established clinical nursing education systems and routinely host nursing interns from multiple universities. The study was approved by the Ethics Committee of Xinjiang Uygur Autonomous Region People’s Hospital. Written informed consent was obtained from all participants before data collection.

### Inclusion criteria

Participants were included if they met the following criteria: (1) Full-time undergraduate nursing students admitted through national unified recruitment; (2) Aged over 18 years; (3) Had completed at least 4 weeks of clinical internship; (4) Provided informed consent and voluntarily agreed to participate in the study; (5) Were able to complete the questionnaire independently and accurately.

### Exclusion criteria

Students were excluded if they: (1) Had been absent from clinical internship for more than two consecutive weeks due to illness or personal reasons; (2) Had prior clinical work or internship experience.

#### Sample size calculation

The sample size was calculated using a standard deviation of 0.5, a margin of error of 0.05, and a 95% confidence level. Based on these parameters, the minimum required sample size was determined to be 360 participants.

### Instruments

#### Perceived professional benefit questionnaire for nurses

Perceived professional benefit was measured using the Nurses’ Perceived Professional Benefit Questionnaire developed by Hu and Liu for use in the Chinese nursing context. The original instrument contains 33 items across five dimensions: self-growth (8 items), positive nurse–patient relationships (6 items), recognition from family and friends (6 items), positive perception of the profession (7 items), and team belongingness (6 items). Each item is rated on a five-point Likert scale (1 = strongly disagree to 5 = strongly agree), with total scores ranging from 33 to 165; higher scores indicate higher perceived professional benefit (14). In the original development study, the questionnaire demonstrated good psychometric performance, with an overall Cronbach’s α of 0.958 and split-half reliability of 0.933. In the present study, the overall Cronbach’s α for the total scale was 0.815.

#### Nursing clinical teacher effective inventory (NCTEI)

Clinical teaching behaviors were assessed using the NCTEI developed by Knox and Mogan and subsequently reported in the classic work by Mogan and Knox. The instrument contains 48 items grouped into five dimensions: teaching ability/competence (16 items), nursing competence/professionalism (10 items), evaluation (9 items), personality traits (7 items), and interpersonal relationships (6 items). Items are rated on a Likert scale, with higher scores indicating more favorable evaluations of clinical teaching effectiveness (15). Previous psychometric reports have shown that the NCTEI has good reliability and stability, with Cronbach’s α values ranging from 0.79 to 0.92 and test–retest correlations ranging from 0.76 to 0.93, along with acceptable face and content validity. In the present study, the overall Cronbach’s α coefficient for the total NCTEI was 0.960.

#### Cultural adaptation and local validation

The Nurses’ Perceived Professional Benefit Questionnaire was originally developed and validated in the Chinese nursing context; therefore, the original Chinese version was used without additional cultural adaptation. For the NCTEI, a translation and cultural adaptation procedure was conducted using translation–back translation, followed by expert review. A pilot test with 30 eligible nursing interns supported the feasibility and comprehensibility of the Chinese version. In the current sample, the overall Cronbach’s α for the NCTEI was 0.960, and the Cronbach’s α values for its five subscales ranged from 0.769 to 0.812, indicating acceptable to excellent internal consistency.

#### Socio-demographic questionnaire

A self-designed socio-demographic questionnaire was used to collect participants’ general characteristics. The questionnaire was developed by the research team based on the study objectives and relevant literature. It included variables such as gender, only-child status, place of residence, presence of medical professionals in the family, whether the nursing major was chosen voluntarily, student leadership experience, and parental educational attainment.

#### Common method bias

Given that all measures were self-reported, several procedural remedies were applied to reduce potential common method bias, including anonymous data collection, emphasizing honest responses, and adjusting the presentation order of the two questionnaires to reduce response patterns.

#### Sample size calculation

This cross-sectional survey estimated the required sample size based on the precision of a mean estimate (i.e., controlling the half-width of the 95% confidence interval). The sample size was calculated using: *n* = (Zα/2 × SD/E)^2^ where Zα/2 = 1.96 for a 95% confidence level, SD is the anticipated population standard deviation, and E is the allowable margin of error (half-width of the confidence interval). Substituting Zα/2 = 1.96, SD = 0.5 and E = 0.05, the required minimum sample size was: *n* = (1.96 × 0.5/0.05)^2^ ≈ 384. We included 387 valid participants in the final analysis.

### Statistical analysis

All data were entered into and analyzed using IBM SPSS Statistics version 26.0. Descriptive statistics were used to summarize participants’ demographic characteristics, perceived professional benefit scores, and evaluations of teaching behaviors. Continuous variables were presented as means and standard deviations (SD), and categorical variables as frequencies and percentages. Internal consistency of the questionnaires was assessed using Cronbach’s alpha coefficients. Normality of continuous variables was examined using the Shapiro-Wilk test, with *p* > 0.05 indicating approximate normality. Independent-samples t tests or one-way ANOVA were used to compare perceived professional benefit scores across demographic groups. Correlations between perceived professional benefit and clinical teaching behaviors were examined using Pearson’s correlation when both variables were continuous and approximately normally distributed; otherwise, Spearman’s rank correlation was applied. Multiple linear regression was performed to identify factors associated with perceived professional benefit. Multicollinearity was assessed using variance inflation factor (VIF) and tolerance, with VIF < 5 and tolerance >0.20 considered acceptable. A two-tailed *p*-value < 0.05 was considered statistically significant.

## Results

### Demographic characteristics of undergraduate nursing students

A total of 387 undergraduate nursing interns from two tertiary hospitals were included in the final analysis. The demographic characteristics of the participants are presented in Table 1. Among them, 336 (86.8%) were female and 51 (13.2%) were male. A total of 163 students (42.1%) were from one-child families, while 224 (57.9%) were not. Regarding place of residence, 98 students (25.3%) were from urban areas, 104 (26.9%) from township regions, and 185 (47.8%) from rural areas. Approximately one-third of the participants (132, 34.1%) reported having a medical professional in their family. Slightly over half of the students (207, 53.5%) voluntarily chose the nursing major, while 180 (46.5%) did not. In terms of leadership experience, 148 (38.2%) served as student leaders. With respect to parental education level, 144 students (37.2%) reported that their mother or father’s highest education was junior high school or below, 196 (50.6%) reported senior high school or vocational school, and 47 (12.2%) reported college diploma or above.

**TABLE 1:** Demographic characteristics of undergraduate nursing students (*n* = 387).

Characteristics	Numbers	Percentage (%)
Gender		
Male	51	13.2%
Female	336	86.8%
One-child family		
Yes	163	42.1%
No	224	57.9%
Residence		
City	98	25.3%
Rural area	185	47.8%
Township	104	26.9%
Whether there is medical professional in the family		
Yes	132	34.1%
No	255	65.9%
Was the selection of the nursing major voluntary?		
Yes	207	53.5%
No	180	46.5%
Student leader		
Yes	148	38.2%
No	239	61.8%
Mother or father’s highest educational attainment		
Junior high school and below	144	37.2%
Senior high school or secondary vocational school	196	50.6%
College diploma and above	47	12.2%

Values were presented as *n* (percentage, %). Percentages were calculated as (number of participants in each subgroup/total sample size, *n* = 387) × 100%.

### Measurement analysis of undergraduate nursing students’ perceived benefits of nursing profession

Table 2 presents the measurement results of undergraduate nursing students’ perceived professional benefit across five dimensions. The overall perceived professional benefit score was 128.41 ± 21.34, indicating generally positive perceptions among participants. Among the five dimensions, the highest score was observed in self-growth (33.29 ± 5.12), followed by positive career perception (26.95 ± 4.78), team belongingness (24.02 ± 5.06), positive nurse–patient relationships (22.04 ± 4.47), and recognition from family and friends (21.86 ± 4.35). Internal consistency was acceptable for each subscale (Cronbach’s α = 0.779–0.809), and the overall scale showed good reliability (Cronbach’s α = 0.815).

**TABLE 2:** Measurement analysis of undergraduate nursing students’ perceived benefits of nursing profession.

Perceived benefits	Number of items	Score	Cronbach’s α
Self-growth	8	33.29 ± 5.12	0.784
Positive nurse-patient relationships	6	22.04 ± 4.47	0.802
Recognition from family and friends	6	21.86 ± 4.35	0.796
Positive career perception	7	26.95 ± 4.78	0.779
Team belongingness	6	24.02 ± 5.06	0.809
Total	33	128.41 ± 21.34	0.815

Values were presented as mean ± SD. Cronbach’s α coefficients reported here are the internal consistency of the scale in the current study sample. All α coefficients > 0.7 indicate acceptable reliability.

### Impact of demographic characteristics of undergraduate nursing students on their perceived benefits of nursing profession

Table 3 summarizes univariate comparisons of perceived professional benefit across demographic subgroups. Students from non-one-child families and those who voluntarily selected the nursing major had higher total perceived professional benefit scores (both *p* < 0.001). Specifically, non-one-child students scored higher in self-growth (34.81 ± 5.27 vs. 31.76 ± 5.33, *p* < 0.001), positive career perception (27.58 ± 4.77 vs. 26.34 ± 4.84, *p* = 0.003), and team belongingness (25.32 ± 5.20 vs. 22.76 ± 4.87, *p* < 0.001). Students who voluntarily chose nursing also showed higher scores across all five dimensions and the total score (134.27 ± 21.23 vs. 122.15 ± 21.95, *p* < 0.001).

**TABLE 3:** Impact of demographic characteristics of undergraduate nursing students on their perceived benefits of nursing profession.

Characteristics	Self-growth	Positive nurse-patient relationships	Recognition from family and friends	Positive career perception	Team belongingness	Total
Gender						
Male	33.71 ± 5.22	22.35 ± 4.79	21.72 ± 4.41	27.11 ± 4.82	24.33 ± 4.99	129.23 ± 23.06
Female	32.94 ± 5.18	21.76 ± 4.37	21.97 ± 4.49	26.79 ± 4.69	23.72 ± 5.14	127.62 ± 22.15
*P*-values	0.314	0.663	0.711	0.681	0.403	0.357
One-child family						
Yes	31.76 ± 5.33	21.17 ± 4.85	22.13 ± 4.62	26.34 ± 4.84	22.76 ± 4.87	124.46 ± 21.28
No	34.81 ± 5.27	22.91 ± 4.62	21.59 ± 4.39	27.58 ± 4.77	25.32 ± 5.20	131.95 ± 22.03
*P*-values	<0.001	0.128	0.426	0.003	<0.001	<0.001
Residence						
City or township	32.65 ± 5.18	22.74 ± 4.77	22.3 ± 4.47	27.44 ± 4.66	24.29 ± 5.04	129.34 ± 21.52
Rural area	33.92 ± 5.32	21.46 ± 4.52	21.52 ± 4.28	26.51 ± 4.91	23.75 ± 5.06	127.16 ± 21.07
*P*-values	0.163	0.323	0.309	0.099	0.510	0.285
Whether there is medical professional in the family						
Yes	33.56 ± 4.97	22.86 ± 4.66	22.81 ± 4.07	26.76 ± 4.74	24.50 ± 5.11	130.27 ± 21.80
No	33.02 ± 5.25	21.38 ± 4.91	21.05 ± 4.26	27.20 ± 4.69	23.61 ± 5.07	127.09 ± 22.07
*P*-values	0.648	0.175	0.005	0.381	0.244	0.178
Was the selection of the nursing major voluntary?						
Yes	35.02 ± 0.519	23.35 ± 4.52	22.67 ± 4.49	27.88 ± 4.85	25.47 ± 5.25	134.27 ± 21.23
No	31.56 ± 0.504	20.69 ± 4.33	21.08 ± 4.40	26.01 ± 4.78	22.59 ± 5.11	122.15 ± 21.95
*P*-values	<0.001	<0.001	0.007	<0.001	<0.001	<0.001
Student leader						
Yes	33.93 ± 5.44	22.14 ± 4.73	22.27 ± 4.52	27.31 ± 4.80	24.39 ± 4.98	130.04 ± 22.09
No	32.69 ± 4.88	21.95 ± 4.80	21.49 ± 4.39	26.65 ± 4.73	23.66 ± 5.07	126.74 ± 1.18
*P*-values	0.122	0.708	0.274	0.262	0.229	0.152
Mother or father’s highest educational attainment						
Junior high school and below	33.16 ± 5.15	21.56 ± 4.91	22.04 ± 4.18	26.58 ± 4.91	23.59 ± 5.03	127.11 ± 21.57
Senior high school or secondary vocational school and above	33.42 ± 5.09	22.58 ± 4.84	21.47 ± 4.33	27.31 ± 4.77	24.40 ± 5.09	129.02 ± 21.22
*P*-values	0.741	0.294	0.315	0.407	0.218	0.244

Values were presented as mean ± SD. Statistical comparisons were performed using the Unpaired *t*-test with Welch’s correction for normally distributed data and the Mann-Whitney U test for non-normally distributed data. Normality was assessed via Shapiro-Wilk test.

Other demographic variables (gender, residence, family medical background, student leadership status, and parental education level) were not significantly associated with the total perceived professional benefit score (*p* > 0.05 for all). However, participants with medical professionals in their family reported higher scores on recognition from family and friends (22.81 ± 4.07 vs. 21.05 ± 4.26, *p* = 0.005).

### Measurement analysis of undergraduate nursing students’ nursing clinical teacher effective inventory

Table 4 presents the measurement results of the NCTEI, which assesses nursing students’ perceived importance of clinical instructors’ teaching behaviors across five dimensions. The total NCTEI score was 175.42 ± 28.75. Among the five dimensions, teaching competence received the highest score (61.17 ± 10.52), followed by professionalism (34.80 ± 7.88), evaluation of nursing students (32.06 ± 5.39), personal traits (26.74 ± 5.44), and interpersonal relationships (22.25 ± 4.63). Subscale internal consistency was acceptable (Cronbach’s α = 0.769–0.812), and the overall scale reliability was 0.796.

**TABLE 4:** Measurement analysis of undergraduate nursing students’ nursing clinical teacher effective inventory.

NCTEI	Number of items	Score	Cronbach’s α
Teaching competence	16	61.17 ± 10.52	0.769
Professionalism	10	34.80 ± 7.88	0.801
Evaluation of nursing students	9	32.06 ± 5.39	0.785
Personal traits	7	26.74 ± 5.44	0.812
Interpersonal relationships	6	22.25 ± 4.63	0.794
Total	48	175.42 ± 28.75	0.796

Values were presented as mean ± SD. Cronbach’s α coefficients reported here are the internal consistency of the scale in the current study sample. All α coefficients > 0.7 indicate acceptable reliability.

### Correlation between perceived benefits of nursing profession and nursing clinical teacher effective inventory in undergraduate nursing students

Table 5 presents correlations between perceived professional benefit and NCTEI dimensions. Pearson or Spearman correlation coefficients were applied according to normality assumptions. Perceived professional benefit was positively correlated with all NCTEI dimensions (*p* < 0.001 for all). The total perceived professional benefit score showed the strongest correlation with interpersonal relationships (*r* = 0.583), followed by personal traits (*r* = 0.529), teaching competence (*r* = 0.523), professionalism (*r* = 0.473), and evaluation of nursing students (*r* = 0.458). Among perceived professional benefit dimensions, positive career perception and team belongingness showed relatively stronger correlations with teaching behavior dimensions.

**TABLE 5:** Correlation between perceived benefits of nursing profession and nursing clinical teacher effective inventory in undergraduate nursing students.

Perceived benefits	Teaching competence	Professionalism	Evaluation of nursing students	Personal traits	Interpersonal relationships	Total
Self-growth	0.516***	0.452***	0.427***	0.534***	0.472***	0.469***
Positive nurse-patient relationships	0.447***	0.487***	0.388***	0.557***	0.542***	0.492***
Recognition from family and friends	0.461***	0.392***	0.403***	0.486***	0.606***	0.478***
Positive career perception	0.548***	0.511***	0.479***	0.581***	0.534***	0.523***
Team belongingness	0.481***	0.496***	0.511***	0.505***	0.611***	0.541***
Total	0.523***	0.473***	0.458***	0.529***	0.583***	0.537***

Correlation coefficients were calculated using Pearson correlation analysis (for bivariate normally distributed continuous variables) or Spearman rank correlation analysis (for non-normally distributed variables or ordinal variables). Normality was evaluated by Shapiro-Wilk test. ***P < 0.001.

### Multiple linear regression analysis of the total score of perceived benefits of nursing profession in undergraduate nursing students

Table 6 presents the results of the multiple linear regression analysis examining factors associated with the total perceived professional benefit score among undergraduate nursing students. The dependent variable was the total score of the perceived professional benefit questionnaire. Independent variables entered into the final model included one-child family status, voluntary selection of the nursing major, and teachers’ personal traits. Categorical variables were coded as follows: one-child family status (0 = non-one-child, 1 = one-child) and voluntary selection of the nursing major (0 = no, 1 = yes). The overall regression model was statistically significant (F = 58.951, *p* < 0.001), and explained 33.5% of the variance in perceived professional benefit scores (R^2^ = 0.335; adjusted R^2^ = 0.326), indicating a moderate explanatory power. Regression diagnostics showed no evidence of problematic multicollinearity (VIF < 5; tolerance > 0.20), and the residuals were approximately normally distributed without obvious heteroscedasticity.

**TABLE 6:** Multiple linear regression analysis of factors associated with the total perceived professional benefit score among undergraduate nursing students.

Variables	β (95% CIs)	SE	β_std	*t*	*P*
One-child family	−2.522 (−4.101 to −0.943)	0.810	−0.237	−3.114	0.002
Voluntary selection of the nursing major	3.374 (1.897 to 4.851)	0.824	0.416	4.095	<0.001
Teacher’s personal traits	4.782 (3.551 to 6.013)	0.839	0.552	5.699	<0.001

R^2^ = 0.335, adjusted R^2^ = 0.326, F = 58.951. β, unstandardized regression coefficient; β*_*std, standardized regression coefficient; SE, standard error; CI, confidence interval.

Among the included predictors, three variables remained statistically significant. One-child family status was negatively associated with the total perceived professional benefit score (β = −2.522, SE = 0.810, *t* = −3.114, p = 0.002, 95% CI: −4.101 to −0.943), indicating that students from one-child families had lower perceived professional benefit scores than those from non-one-child families after adjustment for other variables. Voluntary selection of the nursing major was positively associated with perceived professional benefit (β = 3.374, SE = 0.824, *t* = 4.095, *p* < 0.001, 95% CI: 1.897 to 4.851), suggesting that students who chose nursing voluntarily tended to report higher professional benefit scores. Teachers’ personal traits also showed a significant positive association with perceived professional benefit (β = 4.782, SE = 0.839, *t* = 5.699, *p* < 0.001, 95% CI: 3.551 to 6.013), and had the largest standardized regression coefficient (β_std = 0.552), indicating that it was the strongest predictor in the final model.

## Discussion

This study investigated the current status of perceived professional benefits among undergraduate nursing students and explored their relationship with clinical nursing teachers’ teaching behaviors. The findings provide insights into the associations between students’ personal background, the clinical educational environment, and perceived professional benefit during internship, but do not allow causal or directional inferences. Accordingly, we interpret the results as correlational and avoid attributing explanatory or influential effects.

Overall, nursing students reported moderate to high levels of perceived professional benefit, with self-growth, positive career perception, and team belongingness being the most highly rated dimensions. This aligns with previous studies showing that clinical internship experiences often foster students’ personal development and strengthen their sense of belonging to the healthcare team (16, 17). In contrast, recognition from family and friends and positive nurse–patient relationships received slightly lower scores, suggesting that external validation and relational satisfaction may be less consistently experienced during early clinical training. Notably, these dimension-level differences may also reflect contextual factors in internship settings (e.g., high workload and limited time for reflective communication), which warrant further exploration.

Demographic factors were associated with students’ professional perceptions. Notably, non-one-child students reported significantly higher total scores in perceived professional benefit than their one-child counterparts. This finding may reflect underlying psychosocial differences: students from non-one-child families may be more accustomed to sharing responsibilities, adapting to collaborative environments, and developing interpersonal resilience—traits that align closely with the demands of clinical nursing practice. In contrast, only-child students may experience greater psychological pressure and less exposure to group dynamics prior to clinical engagement, which could affect their integration into the nursing profession. However, alternative explanations are possible (e.g., differences in family expectations, social support, or coping styles), and longitudinal studies are needed to clarify how these factors relate to changes in perceived professional benefit over time.

Voluntary selection of the nursing major also was associated with perceived professional benefit. Students who had actively chosen to study nursing, rather than being passively assigned to the discipline, reported significantly higher scores across all dimensions. This underscores the importance of career autonomy and intrinsic motivation. When students perceive nursing as aligned with their interests and values, they are more likely to internalize the profession’s significance and develop a positive professional identity (18, 19). These findings highlight the need for educational institutions and policy-makers to improve career counseling systems and ensure students have adequate information and support when selecting their academic pathways. At the same time, some studies have reported weaker or non-significant links between major choice motivation and professional-related outcomes. For example, Akince et al. found that the reason for choosing the nursing department was not significantly associated with practice readiness, humanistic practice ability, or lifelong learning in senior nursing students (20). These inconsistencies suggest that the relationship between major-choice motivation and professional outcomes may vary across educational stages, outcome constructs, and institutional contexts (21).

A core focus of this study was the role of clinical teaching behaviors. The results showed that nursing students highly valued effective teaching behaviors, especially in dimensions related to teaching competence and professionalism. These findings are consistent with prior studies indicating that students perceive clinical instructors not only as knowledge transmitters but also as role models whose behaviors and attitudes shape students’ understanding of professional standards (22, 23). Interestingly, personal traits and interpersonal relationships of instructors also received strong evaluations and demonstrated robust correlations with students’ perceived professional benefits. These results suggest that beyond technical and pedagogical skills, the emotional support and relational quality offered by instructors are closely associated with students’ engagement with the profession. This pattern aligns with a humanistic and learner-centered education framework, which emphasizes autonomy, empowerment, collaboration, engagement, respect, and the facilitation of students’ holistic growth within the learning environment (24).

The correlation analysis confirmed that all five dimensions of the teaching behavior inventory were significantly and positively associated with students’ perceived benefits of the nursing profession. The strongest correlations were observed between personal traits and various dimensions of perceived benefit, particularly positive career perception and team belongingness. These findings highlight the value of emotionally intelligent, communicative, and empathetic instructors in clinical settings. Humanistic teaching styles that prioritize respect, encouragement, and individualized support may be linked to a more affirming and meaningful learning experience for nursing students, thereby supporting their professional self-concept and career commitment. Nevertheless, prior evidence is not entirely uniform. Taylan et al. reported that only some clinical instructor caring subdomains were linked to specific future-oriented professional outcomes, while it was found that interpersonal relations showed only a weak association with students’ motivation for learning compared with other teaching-behavior domains (25). Such variation may reflect differences in measurement focus, student seniority, and clinical training context (22).

The results of the multiple linear regression analysis further identified variables independently associated with perceived professional benefit. Voluntary selection of nursing as a major, being from a non-one-child family, and high ratings of instructors’ personal traits were significantly associated with higher perceived professional benefit scores. Among these, the impact of clinical teachers’ personal traits was particularly notable, accounting for the highest standardized coefficient in the model. This finding emphasizes the need for professional development programs that not only improve teaching skills but also enhance the interpersonal and mentoring capacities of nursing educators. Training in areas such as communication, feedback delivery, emotional intelligence, and student-centered pedagogy could substantially improve the clinical learning experience and, in turn, students’ attitudes toward nursing (26). Importantly, the model explained approximately one-third of the variance (R^2^ = 0.335), indicating that a substantial proportion of variability (66.5%) remains unexplained and may relate to factors not measured in this study (e.g., rotation specialty, internship duration, preceptor workload, perceived stress, and organizational climate).

These findings also offer important implications for addressing the ongoing global nursing shortage. Enhancing students’ perceived professional benefits may be relevant to retention and attrition in the nursing workforce. Given the high turnover rates and dissatisfaction among practicing nurses, investing in the emotional and professional formation of nursing students represents a proactive approach to long-term workforce sustainability. However, because this study was cross-sectional and conducted in two tertiary hospitals within one region, the practical implications should be interpreted cautiously, and multi-center longitudinal research is warranted to test whether improving specific teaching behaviors corresponds to changes in perceived professional benefit over time.

Our findings should be interpreted within the cultural and educational context of China. Family structure in China has been shaped by historical demographic policies, and only-child status may be associated with distinct parenting styles, family expectations, and social support patterns, which could influence nursing students’ professional perceptions during internship. Moreover, the organization of undergraduate nursing education and clinical training in China—including the structure of hospital-based internships, preceptor roles, and teaching–learning dynamics—may differ from those in other countries or healthcare systems. Therefore, the observed associations may not be fully generalizable to settings with different family structures, cultural norms, or nursing education models. Future multi-center studies across diverse regions and cross-cultural comparisons are warranted to test the robustness of these relationships in different educational and cultural contexts.

Several limitations of this study should be more strongly emphasized. First, the cross-sectional design precludes causal inference; the observed relationships should be interpreted as associations only, and longitudinal or experimental studies are needed to examine changes in perceived professional benefit over time and to clarify temporal ordering. Second, all measures were collected via self-report questionnaires, which may introduce common method variance and response bias (e.g., social desirability and recall bias), even though validated instruments and anonymity procedures were used. Third, the sample was recruited from only two tertiary (Grade A) hospitals in one region of China, which may limit generalizability to nursing interns in other provinces, hospital levels (e.g., secondary hospitals or community settings), or different training models. Finally, the findings may be culturally specific: family structure (e.g., only-child status) and the organization of nursing education and clinical teaching in China may differ from other countries, which could affect the applicability of the results across cultural and educational contexts. Future multi-center, cross-cultural, and longitudinal studies incorporating broader institutional and contextual variables are warranted.

Despite these limitations, this study provides robust evidence that clinical teaching behaviors-especially those reflecting positive personal traits and relational engagement-are closely linked to nursing students’ perception of professional benefit. It also confirms the importance of intrinsic motivation and family background in shaping students’ professional attitudes. To promote a more engaged and committed nursing workforce, educational institutions should implement multifaceted strategies that include optimizing clinical teaching practices, enhancing career guidance, and providing supportive learning environments.

## Conclusion

This cross-sectional study found that undergraduate nursing interns reported moderate-to-high perceived professional benefit and that perceived professional benefit was positively associated with students’ evaluations of clinical teaching behaviors, particularly teachers’ personal traits. Only-child status and voluntary selection of the nursing major were also independently associated with perceived professional benefit. These findings highlight the importance of supportive, learner-centered clinical teaching and informed career guidance during internship. Further longitudinal and multi-center studies are warranted to confirm these associations and explore additional contributing factors.

## Data Availability

The raw data supporting the conclusions of this article will be made available by the authors, without undue reservation.
